# Psychiatric Comorbidity in Hidradenitis Suppurativa—A Large-Scale Retrospective Cohort Study

**DOI:** 10.3390/jcm15134982

**Published:** 2026-06-26

**Authors:** Beata Jastrząb-Miśkiewicz, Jacek C. Szepietowski, Piotr K. Krajewski

**Affiliations:** Division of Dermatology, Venereology and Clinical Immunology, Faculty of Medicine, Wroclaw University of Science and Technology, 50-377 Wroclaw, Poland; jacek.szepietowski@pwr.edu.pl (J.C.S.);

**Keywords:** hidradenitis suppurativa, psychiatric comorbidity, depression, anxiety, suicidality, substance use disorder, TriNetX, propensity score matching, sex differences, real-world data

## Abstract

**Background/Objectives**: Hidradenitis suppurativa (HS) is a chronic inflammatory skin disease associated with psychiatric burden, but longitudinal data on incident psychiatric outcomes remain limited. This study aimed to evaluate incident psychiatric disorders in adults with HS compared with matched non-HS controls and to assess sex-specific risk. **Methods**: We conducted a retrospective propensity score–matched cohort study using the TriNetX Global Collaborative Network. Adults with at least two HS diagnoses and no prior psychiatric diagnosis were compared with non-HS controls with repeated general health examination encounters and no psychiatric history. Time-to-event analyses estimated hazard ratios (HRs) with 95% confidence intervals (CIs). Sensitivity analyses used a 30-day lag and restriction to the most recent 5-year period. **Results**: After matching, 37,964 pairs were retained for the primary individual-outcome analysis. Median follow-up was shorter in the HS cohort than in matched controls (844 vs. 1505 days). HS was associated with increased risk of any psychiatric disorder (12.3% vs. 5.8%; HR 3.17, 95% CI 3.01–3.34) and severe psychiatric illness (0.6% vs. 0.1%; HR 6.70, 95% CI 4.77–9.41). Elevated risks were observed for bipolar/manic disorders, personality disorders, substance use disorders, psychotic disorders, suicidal ideation, depression, eating disorders, anxiety, and insomnia/parasomnia. Women had higher hazards of depression and anxiety, whereas men had higher hazards of substance use disorders; insomnia/parasomnia showed a nominal association with higher hazard in men. **Conclusions**: In this observational EHR-based study, HS was associated with broad incident psychiatric morbidity. These findings support consideration of proactive mental health assessment and integrated dermatologic–psychiatric care in patients with HS.

## 1. Introduction

Hidradenitis suppurativa (HS) is a chronic, inflammatory skin disorder associated with significant physical and psychosocial morbidity [1]. Its recurrent, painful lesions and disfiguring scarring often affect young adults in their most socially and professionally formative years, limiting daily functioning and social participation [1,2]. The burden of HS extends well beyond skin symptoms. Recent studies highlight profound effects on employment, intimate relationships, and mental well-being [3,4]. Patients with HS are disproportionately affected by psychiatric disorders, including depression, anxiety, substance use, and suicidality [5,6,7,8]. These challenges have led to growing calls for integrating psychodermatology into clinical care for HS, as emphasized in the recent literature [9,10].

HS has an estimated global prevalence between 0.67% and 1.46%, with a clear female predominance and typical onset in early adulthood [5,11,12]. Diagnosis is often delayed by several years, further exacerbating physical suffering and psychosocial distress [6,13]. The chronic, relapsing course of the disease contributes to frustration and hopelessness among patients [14].

Beyond psychosocial contributors, biological mechanisms may also represent plausible contributors to psychiatric risk in HS. Systemic inflammation is increasingly recognized as a contributing factor in the development of psychiatric disorders. Elevated levels of pro-inflammatory cytokines in HS, such as TNF-α, IL-6, and IL-17, may affect neuroendocrine and neurotransmitter systems, potentially contributing to depression and anxiety [10,15,16,17,18]. Recent studies underscore shared immune pathways between HS and psychiatric conditions, supporting the biological plausibility of an inflammation–mental health link [10,19]. However, inflammatory biomarkers and mechanistic pathways were not directly assessed in the present EHR-based study. The chronic nature and psychosocial burden of HS may further exacerbate psychiatric vulnerability [20,21].

While previous studies have established an association between HS and psychiatric disorders, most are limited by small sample sizes, cross-sectional design, and methodological heterogeneity [1,10,13,14,22,23]. Although recent real-world data have begun to characterize sex-specific differences in selected psychiatric diagnoses and psychotropic medication use among patients with HS [24], longitudinal data on incident psychiatric morbidity across broad psychiatric outcome categories remain limited. In light of these limitations, we conducted a large-scale, matched-control cohort study using electronic health record data to investigate the prevalence and cumulative risk of psychiatric disorders in patients with HS and to explore sex-specific patterns of psychiatric risk. We hypothesized that HS would be associated with an increased risk of incident psychiatric morbidity compared with matched non-HS controls and that psychiatric risk profiles would differ between female and male patients with HS.

## 2. Materials and Methods

### 2.1. Ethics Statement

This research is a secondary analysis of pre-existing, de-identified data and does not involve any direct interaction or intervention with human participants. All data comply with the de-identification criteria outlined in Section §164.514(a) of the HIPAA Privacy Rule. The TriNetX network operates under a comprehensive governance and security framework, serving as a federated real-world data platform that complies with the Health Insurance Portability and Accountability Act (HIPAA), the General Data Protection Regulation (GDPR), and the Lei Geral de Proteção de Dados (LGPD). Consequently, the present study was exempt from Institutional Review Board (IRB) oversight.

### 2.2. Study Design and Database

This was a retrospective propensity score–matched (PSM) cohort study using the TriNetX Global Collaborative Network. All cohort queries and analyses were executed and exported from TriNetX on 1 May 2026, which served as the data-extraction date for this study. Because TriNetX is continuously updated, no static public dataset version was available. The primary objective was to compare the cumulative risk of incident psychiatric disorders among patients diagnosed with HS with those of PSM non-HS controls.

All analyses were conducted using the TriNetX Global Collaborative Network, a federated international real-world data network that provides access to de-identified electronic health records from participating healthcare organizations (HCOs). At the time of data extraction, the queried network included 172 HCOs across multiple countries and healthcare systems. Contributing HCOs are heterogeneous and may include academic medical centers, hospital systems, specialty care providers, and other healthcare institutions. Available data include diagnoses, procedures, medications, laboratory results, and other structured EHR-derived information harmonized within the TriNetX platform.

### 2.3. Study Population

Adults aged 18 years or older with recorded male or female sex were eligible for inclusion. In the primary analysis, the HS cohort comprised patients with at least two recorded diagnoses of HS, defined by the ICD-10-CM code L73.2. Patients with any documented psychiatric diagnosis before or on the index date were excluded using ICD-10-CM codes F01–F99.

The non-HS comparator cohort consisted of adults without any recorded diagnosis of HS who had at least two encounters coded as general health examination without complaint, suspected, or reported diagnosis (ICD-10-CM Z00), including at least one Z00 encounter occurring 3 to 5 years after a qualifying Z00 encounter. Controls with any psychiatric diagnosis before or on the index date were excluded.

Sex-specific HS subcohorts were created to compare psychiatric outcomes between female and male patients with HS.

### 2.4. Outcomes

The primary outcome was incident diagnosis of any psychiatric disorder, defined as ICD-10-CM codes F01–F99. This broad composite outcome was used to estimate overall incident psychiatric morbidity. Secondary psychiatric outcomes included individual psychiatric diagnostic categories: depression (F32, F33, F34.1), anxiety disorders (F40, F41, F43), bipolar disorder and manic episodes (F30, F31), psychotic disorders (F20–F29), eating disorders (F50), personality disorders (F60–F69), insomnia and parasomnia (G47, F51), suicidal ideation (R45.851), personal history of suicidal behavior (Z91.51), suicide attempts (T14.91), substance use disorders (F10–F19), and self-harm (X71–X83, R45.88). To further characterize clinically severe psychiatric morbidity, we also evaluated a prespecified severe psychiatric illness composite, defined as psychotic disorders, manic episodes, or bipolar disorder (F20–F29, F30, F31). This operational composite was intended to capture psychotic-spectrum or manic/bipolar presentations and was not intended to encompass all severe psychiatric conditions.

Patients with the outcome of interest recorded before the beginning of the analysis time window were excluded from the corresponding outcome-specific analysis. Outcomes with insufficient event counts to generate reliable estimates were not analyzed as standalone endpoints and are described qualitatively where applicable.

### 2.5. Covariates

For the HS versus non-HS comparison, propensity score matching was performed using demographic and baseline clinical characteristics recorded before or at the index date. Matching variables included age at index, sex, race and ethnicity categories, tobacco use (Z72.0), personal history of nicotine dependence (Z87.891), Crohn disease (K50), ulcerative colitis (K51), overweight and obesity (E66), diabetes mellitus (E08–E13), pain, not elsewhere classified (G89), essential hypertension (I10), and chronic kidney disease (N18).

Propensity score matching was conducted within the TriNetX platform using nearest-neighbor greedy matching without replacement and the platform’s default caliper of 0.10 standard deviations of the propensity score. Covariate balance before and after matching was assessed using standardized mean differences, with values below 0.1 indicating adequate balance.

For sex-specific analyses within the HS cohort, female and male patients with HS were matched on age at index, race and ethnicity categories, and the same baseline clinical characteristics. Sex was treated as the exposure variable in these analyses.

### 2.6. Sensitivity Analyses

The primary analysis (S1) required at least two recorded HS diagnoses and at least two qualifying Z00 encounters for controls, with psychiatric outcomes assessed from 1 day after the index date.

Two additional analyses were conducted under alternative analytic specifications. For each analysis, cohort eligibility criteria were applied first, followed by independent 1:1 PSM within TriNetX. The S2 analysis used a 30-day post-index lag period, excluding psychiatric outcomes recorded during the first month after cohort entry. This lag was used to reduce the influence of psychiatric diagnoses recorded immediately after cohort entry, which may reflect early detection of previously unrecorded morbidity, intensified healthcare contact, or surveillance bias rather than newly developing psychiatric disease. The S3 analysis restricted cohort entry to the most recent 5-year period.

### 2.7. Statistical Analysis

All analyses were conducted using the TriNetX Analytics platform. Baseline characteristics were summarized using means with standard deviations, medians with interquartile ranges, or counts and percentages, as appropriate. Between-group balance before and after propensity score matching was assessed using standardized mean differences, with values below 0.1 indicating adequate balance.

In matched cohorts, risk analyses were performed for each outcome, excluding patients with that outcome recorded before the start of the analysis window. Absolute risks, risk differences, risk ratios, odds ratios, and corresponding 95% confidence intervals were calculated within the TriNetX platform. Time-to-event analyses were performed using Kaplan–Meier survival analysis, with censoring after the last recorded fact in the patient’s electronic health record. Between-group differences were assessed using log-rank tests.

Hazard ratios (HRs) with 95% confidence intervals were estimated using Cox proportional hazards models. The proportional hazards assumption was formally assessed using the TriNetX proportionality test for each Cox model. When the proportional hazards assumption was not fully supported, HRs were interpreted as average relative hazards over the observed follow-up period rather than as constant effects across time.

Results were reported for the primary analysis (S1) and both sensitivity analyses (S2, S3) described above. Additional analyses compared psychiatric outcomes between female and male patients with HS and used multivariable Cox proportional hazards modeling to evaluate predictors of incident psychiatric disorders within the HS cohort. Nominal two-sided *p*-values are reported. To account for multiple testing across prespecified psychiatric outcomes, Bonferroni correction was applied separately within each analysis family by dividing α = 0.05 by the number of outcome comparisons in that family. Findings not meeting the Bonferroni-adjusted threshold were interpreted as nominal or exploratory.

## 3. Results

### 3.1. Cohort Characteristics and Matching

After outcome-specific exclusions and 1:1 PSM, the primary S1 analysis included 37,964 patients with HS and 37,964 matched non-HS controls. The median follow-up time after matching was 844 days in the HS cohort and 1505 days in the control cohort.

Formal assessment of the proportional hazards assumption using the TriNetX proportionality test showed evidence of non-proportionality for the primary composite outcome of any psychiatric disorder (χ^2^ = 138.958, *p* < 0.001) and for severe psychiatric illness (χ^2^ = 24.406, *p* < 0.001), as well as for several individual outcomes. Therefore, Cox-derived HRs were interpreted as average relative hazards over the observed follow-up period rather than as constant effects across time. Full proportionality test results for the primary and sensitivity analyses are provided in Supplementary Table S1.

After independent matching, S2 included 40,531 matched pairs, and S3 included 46,313 matched pairs. In the sex-specific HS analysis, 10,119 female and 10,119 male patients with HS were retained after PSM.

A detailed summary of baseline characteristics and matching quality for the primary analysis is presented in Table 1. Data for the sensitivity and sex-specific analyses are provided in Supplementary Table S2.

### 3.2. Cumulative Risk of Psychiatric Disorders in HS Patients Compared to Controls

**Figure 1:**
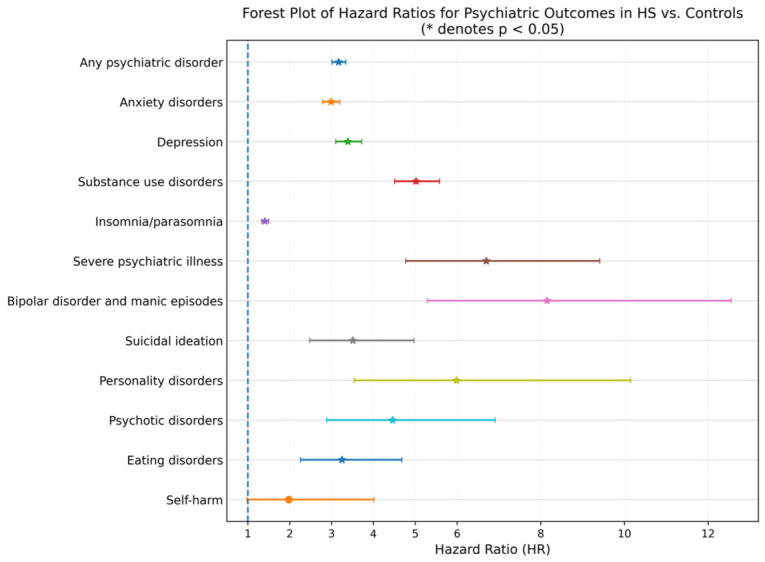
Forest plot of HR of psychiatric outcomes in the HS group versus controls. The vertical dashed line indicates HR = 1.0. (Different colors are used only to distinguish the outcomes and have no additional clinical meaning).

In the primary S1 analysis, patients with HS had a significantly higher cumulative risk of incident psychiatric disorders than matched non-HS controls. HS was associated with an increased risk of any psychiatric disorder and severe psychiatric illness. Among individual outcomes, the strongest associations were observed for bipolar disorder and manic episodes, personality disorders, substance use disorders, psychotic disorders, suicidal ideation, depression, eating disorders, and anxiety disorders. Insomnia and parasomnia showed a more modest association in time-to-event analysis. Self-harm was not significantly increased in the primary analysis, showed a nominal association in the 30-day lag analysis, and was not reported in the most recent 5-year analysis because TriNetX did not generate a reliable estimate for this low-event outcome. Suicide attempts and personal history of suicidal behavior were not analyzed as standalone endpoints because of insufficient event counts. Detailed HRs are presented in Table 2 and Figure 1.

The separately matched sensitivity analyses conducted under alternative analytic specifications showed directionally consistent findings. In the 30-day post-index lag analysis (S2), the association with any psychiatric disorder remained significant (HR 3.03, 95% CI 2.89–3.19, *p* ≤ 0.001). Similarly, in the analysis restricted to patients indexed within the most recent 5-year period (S3), the association persisted with comparable magnitude (HR 3.11, 95% CI 2.97–3.25, *p* ≤ 0.001). Detailed HRs for all sensitivity analyses are provided in Table 2, while event counts, denominators, and absolute risks are presented in Supplementary Table S3.

### 3.3. Sex-Stratified Analysis in HS Patients

**Figure 2:**
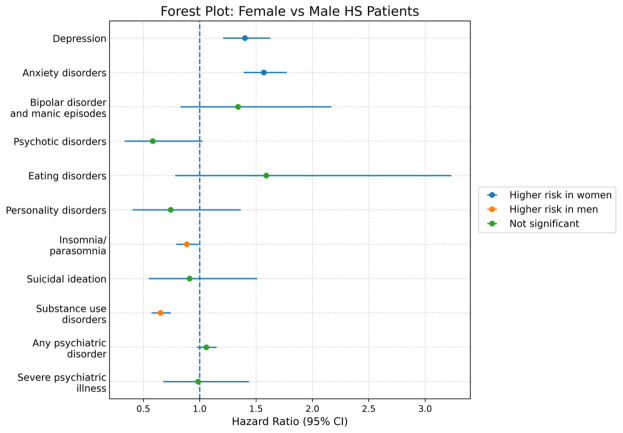
Sex-specific differences in incident psychiatric outcomes among patients with HS. Hazard ratios compare female versus male patients with HS; values below 1.0 indicate a higher hazard in male patients and values above 1.0 indicate a higher hazard in female patients. Associations were interpreted according to the Bonferroni-adjusted significance threshold; insomnia/parasomnia showed only a nominal association with a higher hazard in male patients. The vertical dashed line indicates HR = 1.0.

In sex-stratified analyses, female patients with HS had higher hazards of depression and anxiety disorders, whereas male patients had higher hazards of substance use disorders. Insomnia/parasomnia showed a nominal association with a higher risk in male patients but did not meet the Bonferroni-adjusted significance threshold. No significant sex-based differences were observed for bipolar disorder and manic episodes, eating disorders, personality disorders, suicidal ideation, any psychiatric disorder, or severe psychiatric illness. Psychotic disorders showed a non-significant trend toward higher risk in male patients. Detailed estimates are presented in Table 3 and Figure 2.

### 3.4. Predictors of Psychiatric Disorder Risk in HS (Cox Model)

In the multivariable Cox model, overweight and obesity, pain, and emergency department services were associated with a higher risk of incident psychiatric disorder. Female sex and age at index were also statistically significant predictors; however, the effect size for age was modest on a per-year scale. Prior recorded adalimumab exposure was included as a static treatment-related covariate and was associated with a lower observed hazard of incident psychiatric disorder. This estimate should be interpreted descriptively, as adalimumab was not modeled as a time-varying exposure.

Skin/subcutaneous tissue surgical procedures were not significantly associated with psychiatric risk. Detailed estimates are presented in Table 4.

## 4. Discussion

This large-scale, propensity score–matched cohort study demonstrates that patients with HS are at a markedly increased risk of developing psychiatric disorders compared with non-HS controls. These associations remained directionally consistent across separately matched sensitivity analyses. Clinically, the persistence of elevated hazards after excluding diagnoses recorded during the first 30 days and after restricting the analysis to the most recent 5-year period suggests that the observed psychiatric burden was not driven solely by immediate post-index detection or by older historical data.

The strongest associations were observed for bipolar disorder and manic episodes, severe psychiatric illness, personality disorders, substance use disorders, and psychotic disorders, with consistently elevated hazards also observed for suicidal ideation, depression, eating disorders, and anxiety disorders. These results are consistent with clinical and cohort-level evidence demonstrating elevated rates of emotional distress, anxiety, and depression among individuals with HS, including pediatric populations [2,25,26]. The increased risk of suicidal ideation observed in our cohort corroborates population-based studies reporting excess suicidality among patients with HS [27] and meta-analyses confirming increased suicidality risk [6,28,29].

Importantly, the psychological burden observed in HS should be interpreted within the broader context of psychodermatology and not as a phenomenon unique to HS. Increased rates of psychiatric disorders have been reported in several chronic inflammatory diseases. In psoriasis, a systematic review of the literature revealed a significant burden of depression, with prevalence estimates varying widely across studies and generally higher among patients with more severe disease [30]. In atopic dermatitis, a systematic review and meta-analysis found significant associations with stress, depression, anxiety, and suicidal ideation [31]. Similarly, vitiligo is associated with various comorbid psychosocial disorders, including depression, anxiety, stigma, sleep disturbances, relationship difficulties, and avoidance behaviors [32]. In alopecia areata, a systematic review and meta-analysis found higher levels of anxiety and depression, reduced quality of life, and psychosocial outcomes often comparable to those seen in other dermatological conditions [33]. Therefore, although inflammatory and immunological mechanisms remain biologically plausible psychiatric risk factors in HS, these findings should be considered multifactorial and hypothesis-generating. They may reflect the overlapping effects of chronic visible disease, pain, sleep disturbances, stigma, functional impairment, patterns of healthcare contact, and systemic inflammation, rather than mechanisms unique to HS.

Beyond common psychiatric comorbidities such as depression, anxiety, and suicidality, our analysis identified strong associations with bipolar disorder, manic episodes, and psychotic disorders. Previous large-scale and meta-analytic studies likewise documented increased odds of bipolar disorder and schizophrenia in HS [1,5]. A recent TriNetX propensity-matched cohort study focusing on new-onset bipolar disorder found an increased risk among patients with HS compared with controls, particularly among women [34]. In our study, bipolar disorder and manic episodes were also among the strongest HS-associated outcomes. However, the female-versus-male comparison was not significant, likely reflecting differences in comparator structure between studies. These findings suggest that psychiatric morbidity in HS extends beyond internalizing symptoms and may include severe psychiatric illness. However, the particularly high HRs for bipolar and psychotic disorders should be interpreted cautiously, as these outcomes were less frequent than depression or anxiety and were accompanied by wider confidence intervals.

In addition, the estimate for severe psychiatric illness was attenuated after applying the 30-day post-index lag, decreasing from HR 6.70 (95% CI 4.77–9.41) in the primary analysis to HR 4.98 (95% CI 3.74–6.63) in S2. This attenuation suggests that early post-index detection may have contributed to part of the primary association, potentially reflecting surveillance bias or synchronous recognition of previously unrecorded psychiatric morbidity around the time of HS diagnosis or renewed dermatologic evaluation. Nevertheless, the association remained strong and statistically significant after exclusion of events recorded during the first month, indicating that the observed excess risk was not explained solely by immediate post-index detection.

The heterogeneity of psychiatric outcomes observed in our study suggests that mental health burden in HS may arise through multiple and potentially overlapping pathways, including pain, cumulative psychosocial impairment, functional limitations, and healthcare-related factors [3,35]. These findings highlight the importance of longitudinal follow-up and complementary analytical frameworks to capture both common and severe psychiatric outcomes of HS [13].

Our analyses also revealed significant associations with personality and eating disorders, outcomes that remain comparatively underexplored in HS. Because direct evidence for these specific diagnoses is limited, these findings should be interpreted in the broader context of HS symptom burden rather than as evidence of a defined causal pathway. Pain, pruritus, malodor, and suppuration have been associated with impaired quality of life and with domains including sexual distress, anxiety, depression, and sleep, while population-based data have also linked HS with sleep disorders [36,37,38]. Together, these observations support a more comprehensive view of psychiatric vulnerability in HS, encompassing symptom distress, sleep disruption, and psychosocial impairment.

The strong association with substance use disorders also warrants special attention. Chronic pain associated with HS may contribute to opioid exposure and subsequent opioid misuse, as a population-based study found that substance use disorders were more common in HS patients than in controls, and opioids accounted for almost one-third of substance misuse among HS patients [39]. In a separate cohort of opioid-naive patients, HS was associated with a higher risk of incident long-term opioid use, supporting the need for systematic pain assessment and careful monitoring of opioid prescriptions in this population [40]. These findings suggest that findings regarding substance use in HS may partly reflect attempts to treat chronic pain rather than solely independent psychiatric comorbidities.

The coexistence of HS and psychiatric comorbidities has major implications for healthcare utilization and patient care. Psychiatric illness in HS has been linked with greater use of emergency and surgical services, as shown in large population-based studies where comorbid psychiatric disorders predicted more frequent emergency visits, hospitalizations, and procedures [41]. In our multivariable model, prior emergency department use, pain, and overweight or obesity were independently associated with subsequent psychiatric diagnoses, whereas skin/subcutaneous tissue surgical procedures were not significantly associated with psychiatric risk. Psychiatric symptoms, particularly anxiety, depression, and sleep disturbance, may further compound functional impairment and reduce quality of life in patients with HS [26,42].

Treatment-related factors may also be relevant to psychiatric risk.

In our multivariable analysis, recorded adalimumab exposure was associated with a lower observed hazard of incident psychiatric disorder. This finding is consistent with recent TriNetX-based data reporting lower risks of several mental health diagnoses among biologic-treated patients with HS [43] and with trial data suggesting that adalimumab may reduce pain and improve depressive symptoms in HS [44]. However, adalimumab exposure was modeled as a static treatment-related covariate, and biologic therapy is generally reserved for patients with more severe HS, while validated HS severity measures were unavailable in our dataset. Therefore, this association should be interpreted as descriptive and hypothesis-generating rather than as evidence of a direct protective treatment effect.

Despite increasing recognition of psychiatric burden in HS, systematic psychosocial and mental health screening remains infrequent in dermatologic practice [10,13]. Experts increasingly advocate multidisciplinary models integrating dermatology, psychiatry, psychology, and primary care to support early identification and management of psychiatric symptoms in patients with HS [19,45]. Psychological assessment should therefore be incorporated into routine HS evaluation, particularly in patients presenting with pain, sleep disturbance, substance use, social withdrawal, or recurrent emergency department use. Optimized HS management, including surgical intervention when appropriate, may also improve psychosocial well-being, as suggested by post-treatment improvements in quality-of-life and mental health scores [35]. Collectively, these findings underscore that a coordinated, interdisciplinary approach addressing both cutaneous disease and psychological health is essential to optimize outcomes in HS [5,9].

The present study has several limitations. First, its retrospective design based on routinely collected electronic medical records does not allow for causal inferences.

Although PSM reduced measured baseline differences, it balanced only variables captured and coded in the EHR. This is particularly relevant to tobacco use and overweight/obesity, which were identified using ICD-10-CM codes and were recorded less frequently than expected from clinical HS literature, suggesting incomplete coding. Residual confounding related to under-recorded lifestyle, anthropometric, disease severity, socioeconomic, medication-history, and healthcare-seeking factors, therefore, cannot be excluded.

Second, diagnoses were identified using ICD-10 codes, which may lead to incomplete or inaccurate classification of both HS and psychiatric outcomes. Although requiring at least two recorded HS diagnoses was intended to improve specificity, validation by chart review or clinical examination was not possible in this de-identified database. Psychiatric outcomes were also code-based, and outcomes such as self-harm may be underestimated when intent is not clearly documented; therefore, the absence of a statistically significant association with self-harm in the primary analysis should not be interpreted as a definitive absence of risk. In addition, the sex-specific analyses may have been underpowered for less frequent outcomes, as reflected by small event counts and wide confidence intervals; therefore, non-significant sex differences should not be interpreted as evidence of equivalence between female and male patients with HS. Because female and male HS cohorts were matched on baseline demographic and clinical characteristics, these analyses represent covariate-balanced comparisons rather than estimates of unadjusted total sex-related differences in psychiatric burden.

Third, follow-up duration differed between cohorts, with shorter observation in patients with HS than in matched controls. In addition, the non-HS comparator cohort was defined using repeated Z00-coded general health examination encounters, including a subsequent encounter 3 to 5 years after the qualifying encounter. Although this approach was intended to identify non-HS individuals with continued healthcare-system activity, it may have selected controls with more stable longitudinal healthcare engagement and contributed to differential follow-up and selection bias. Cox models with censoring at the last recorded encounter account for unequal observation time, but they do not eliminate potential bias related to differential or informative censoring. Moreover, formal assessment using the TriNetX proportionality test indicated non-proportionality for the primary composite endpoint and several secondary outcomes; therefore, HRs should be interpreted as average relative hazards over the observed follow-up period rather than as constant effects across time. Detection bias also cannot be ruled out, as patients with HS may use healthcare more frequently than controls, increasing the chances of identifying and coding psychiatric diagnoses.

Fourth, the database did not provide detailed information on treatment duration, adherence, switching patterns, previous treatment failures, clinical response, or validated HS severity measures. Moreover, adalimumab exposure was modeled as a static treatment-related covariate rather than as a time-varying exposure and should not be interpreted as representing biologic therapy as a class. The observed association between adalimumab exposure and lower psychiatric risk may reflect better disease control, closer specialist care, selection factors, or time-related bias, including potential immortal time bias, rather than a direct protective effect.

Finally, despite the large international sample, the results may not be fully generalizable to populations outside the healthcare systems represented in the TriNetX network. Because the study did not include active comparator cohorts with other chronic inflammatory dermatologic diseases, it cannot determine whether the observed pattern and magnitude of psychiatric risk are characteristic of HS or reflect a broader psychodermatologic burden associated with chronic visible inflammatory skin disease. Prospective studies incorporating validated measures of HS severity, standardized psychiatric assessments, detailed data on treatment response, and patient-reported outcomes are needed to elucidate mechanisms and identify modifiable risk factors.

## 5. Conclusions

In summary, HS was associated with an increased risk across a broad spectrum of incident psychiatric disorders, including mood, anxiety, substance use, sleep, eating, and personality disorders, as well as suicidal ideation and severe psychiatric illness comprising bipolar/manic and psychotic disorders. These findings reflect the profound biopsychosocial burden of HS and highlight the need for proactive mental health assessment and integrated care models. Multidisciplinary management addressing both dermatologic and psychological dimensions of HS is essential to improving patient well-being and long-term outcomes.

## Figures and Tables

**Table 1:** Baseline characteristics of HS patients and matched non-HS controls before and after PSM.

Variable	HS Before PSM *n* = 38,687	Controls Before PSM *n* = 1,216,050	SMD	HS After PSM *n* = 37,964	Controls After PSM *n* = 37,964	SMD
Age at index, mean (SD), years	34.4 (14.9)	46.6 (22.8)	0.631	34.4 (14.9)	34.9 (15.4)	0.031
Female sex, *n* (%)	28,004 (73.7)	628,968 (53.0)	0.441	27,976 (73.7)	27,983 (73.7)	<0.001
Male sex, *n* (%)	9988 (26.3)	558,401 (47.0)	0.441	9988 (26.3)	9981 (26.3)	<0.001
White race, *n* (%)	14,491 (38.1)	721,897 (60.8)	0.465	14,491 (38.2)	14,430 (38.0)	0.003
Black or African American race, *n* (%)	12,789 (33.7)	136,580 (11.5)	0.550	12,762 (33.6)	12,878 (33.9)	0.006
Other race, *n* (%)	2530 (6.7)	71,517 (6.0)	0.026	2530 (6.7)	2522 (6.6)	0.001
Not Hispanic or Latino ethnicity, *n* (%)	25,238 (66.4)	760,900 (64.1)	0.049	25,213 (66.4)	25,235 (66.5)	0.001
Unknown ethnicity, *n* (%)	8488 (22.3)	349,360 (29.4)	0.162	8487 (22.4)	8560 (22.5)	0.005
Tobacco use (Z72.0), *n* (%)	410 (1.1)	6401 (0.5)	0.060	408 (1.1)	442 (1.2)	0.009
Personal history of nicotine dependence (Z87.891), *n* (%)	1116 (2.9)	68,291 (5.8)	0.138	1116 (2.9)	1048 (2.8)	0.011
Crohn disease (K50), *n* (%)	608 (1.6)	4871 (0.4)	0.120	581 (1.5)	624 (1.6)	0.009
Ulcerative colitis (K51), *n* (%)	334 (0.9)	6871 (0.6)	0.035	324 (0.9)	374 (1.0)	0.014
Overweight and obesity (E66), *n* (%)	8295 (21.8)	202,573 (17.1)	0.121	8285 (21.8)	8235 (21.7)	0.003
Diabetes mellitus (E08–E13), *n* (%)	3470 (9.1)	114,707 (9.7)	0.018	3468 (9.1)	3435 (9.0)	0.003
Pain, not elsewhere classified (G89), *n* (%)	2364 (6.2)	105,635 (8.9)	0.101	2362 (6.2)	2348 (6.2)	0.002
Essential hypertension (I10), *n* (%)	5307 (14.0)	349,088 (29.4)	0.381	5307 (14.0)	5185 (13.7)	0.009
Chronic kidney disease (N18), *n* (%)	795 (2.1)	48,339 (4.1)	0.115	793 (2.1)	723 (1.9)	0.013

Abbreviations: HS—hidradenitis suppurativa; SMD—standardized mean difference; PSM—propensity score matching; SD—standard deviation.

**Table 2:** Primary and sensitivity analyses of incident psychiatric outcomes in patients with HS compared with matched non-HS controls.

Psychiatric Outcome	S1: Primary Analysis HR (95% CI), *p*-Value	S2: 30-Day Post-Index Lag HR (95% CI), *p*-Value	S3: Most Recent 5-Year Analysis HR (95% CI), *p*-Value
Any psychiatric disorder	3.17 (3.01–3.34), *p* < 0.001	3.03 (2.89–3.19), *p* < 0.001	3.11 (2.97–3.25), *p* < 0.001
Anxiety disorders	2.99 (2.79–3.20), *p* < 0.001	2.84 (2.66–3.03), *p* < 0.001	2.93 (2.76–3.12), *p* < 0.001
Depression	3.39 (3.10–3.72), *p* < 0.001	3.24 (2.98–3.53), *p* < 0.001	3.45 (3.18–3.74), *p* < 0.001
Substance use disorders	5.02 (4.51–5.58), *p* < 0.001	5.12 (4.61–5.68), *p* < 0.001	4.99 (4.55–5.48), *p* < 0.001
Insomnia and parasomnia	1.41 (1.33–1.50), *p* < 0.001	1.44 (1.36–1.52), *p* < 0.001	1.46 (1.39–1.54), *p* < 0.001
Severe psychiatric illness	6.70 (4.77–9.41), *p* < 0.001	4.98 (3.74–6.63), *p* < 0.001	5.74 (4.32–7.62), *p* < 0.001
Bipolar disorder and manic episodes	8.15 (5.29–12.55), *p* < 0.001	7.00 (4.70–10.41), *p* < 0.001	6.84 (4.76–9.83), *p* < 0.001
Suicidal ideation	3.51 (2.48–4.97), *p* < 0.001	3.06 (2.23–4.21), *p* < 0.001	4.00 (2.90–5.53), *p* < 0.001
Personality disorders	5.99 (3.54–10.15), *p* < 0.001	5.11 (3.20–8.17), *p* < 0.001	7.14 (4.25–12.00), *p* < 0.001
Psychotic disorders	4.46 (2.88–6.91), *p* < 0.001	3.38 (2.30–4.96), *p* < 0.001	4.15 (2.79–6.19), *p* < 0.001
Eating disorders	3.25 (2.26–4.68), *p* < 0.001	3.38 (2.36–4.83), *p* < 0.001	3.28 (2.32–4.62), *p* < 0.001
Self-harm	1.98 (0.98–4.01), *p* = 0.054	2.41 (1.15–5.04), *p* = 0.016	Not reported

Abbreviations: CI, confidence interval; HR, hazard ratio; HS, hidradenitis suppurativa.

**Table 3:** Sex-specific analysis of incident psychiatric outcomes among patients with HS.

Psychiatric Outcome	Female HS, Events/Patients	Male HS, Events/Patients	Female vs. Male HR (95% CI), *p*-Value	Direction of Association
Any psychiatric disorder	1210/9936	1034/9936	1.06 (0.97–1.15), *p* = 0.186	Not significant
Anxiety disorders	702/10,119	416/10,119	1.57 (1.39–1.77), *p* < 0.001	Higher risk in women
Depression	439/10,119	289/10,119	1.40 (1.21–1.63), *p* < 0.001	Higher risk in women
Substance use disorders	390/10,119	531/10,119	0.65 (0.57–0.74), *p* < 0.001	Higher risk in men
Insomnia and parasomnia	635/9534	621/9199	0.89 (0.79–0.99), *p* = 0.030	Nominally higher hazard in men
Severe psychiatric illness	57/9936	52/9936	0.99 (0.68–1.44), *p* = 0.941	Not significant
Bipolar disorder and manic episodes	41/10,119	28/10,119	1.34 (0.83–2.17), *p* = 0.231	Not significant
Suicidal ideation	30/10,113	30/10,113	0.91 (0.55–1.51), *p* = 0.714	Not significant
Personality disorders	19/10,119	23/10,119	0.74 (0.40–1.36), *p* = 0.334	Not significant
Psychotic disorders	20/10,119	31/10,119	0.58 (0.33–1.02), *p* = 0.057	Non-significant trend toward higher risk in men
Eating disorders	21/10,119	12/10,119	1.59 (0.78–3.23), *p* = 0.196	Not significant

Abbreviations: CI, confidence interval; HR, hazard ratio; HS, hidradenitis suppurativa. Event counts represent crude cumulative counts, whereas HRs were estimated using Cox proportional hazards models and therefore account for event timing and censoring. Associations with nominal *p* < 0.05 that did not meet the Bonferroni-adjusted threshold are described as nominal.

**Table 4:** Multivariable Cox model for predictors of incident psychiatric disorder among patients with HS.

Predictor	HR (95% CI)	*p*-Value	Interpretation
Female sex	1.08 (1.01–1.16)	0.022	Slightly higher risk
Age at index, per year	1.01 (1.00–1.01)	<0.001	Small increase per year
Overweight and obesity	1.31 (1.23–1.40)	<0.001	Higher risk
Pain, not elsewhere classified	1.62 (1.46–1.78)	<0.001	Higher risk
Skin/subcutaneous tissue surgical procedures	1.04 (0.96–1.13)	0.330	Not significant
Emergency department services	1.31 (1.23–1.40)	<0.001	Higher risk
Recorded adalimumab exposure	0.53 (0.42–0.66)	<0.001	Lower observed hazard; interpret cautiously

Abbreviations: CI, confidence interval; HR, hazard ratio; HS, hidradenitis suppurativa.

## Data Availability

Restrictions apply to the availability of these data. The data were obtained from the TriNetX Global Collaborative Network and are available through TriNetX to eligible users under a data use agreement.

## Supplementary Materials

The following supporting information can be downloaded at: https://www.mdpi.com/article/10.3390/jcm15134982/s1; Table S1: Results of the TriNetX proportional hazards assumption tests for Cox models in the primary and sensitivity analyses. Table S2: Baseline characteristics before and after propensity score matching in sensitivity and sex-specific analyses. Table S3: Event counts and absolute risks for incident psychiatric outcomes in the primary and sensitivity analyses.
