# Supplementary material for: Psychiatric Comorbidity in Hidradenitis Suppurativa—A Large-Scale Retrospective Cohort Study
Source: J Clin Med. 2026 Jun 26;15(13):4982. doi: 10.3390/jcm15134982 (PMC13362446; doi:10.3390/jcm15134982)
Supplement: Supplementary file 1 [file jcm-15-04982-s001.zip › jcm-4366685-supplementary.pdf]

Supplementary Materials:

Supplementary Table S1. Results of the TriNetX proportional hazards assumption tests for Cox models in the primary and sensitivity analyses.

Outcome	S1: Primary analysis, χ^2 (p value)	S2: 30-day post-index lag, χ^2 (p value)	S3: Most recent 5-year analysis, χ^2 (p value)
Any psychiatric disorder	138.958, p<0.001	114.629, p<0.001	181.327, p<0.001
Severe psychiatric illness	24.406, p<0.001	13.614, p<0.001	23.053, p<0.001
Depression	19.564, p<0.001	20.520, p<0.001	31.031, p<0.001
Anxiety disorders	60.569, p<0.001	54.292, p<0.001	75.264, p<0.001
Bipolar disorder and manic episodes	7.581, p=0.006	6.705, p=0.010	8.845, p=0.003
Psychotic disorders	21.524, p<0.001	8.244, p=0.004	15.131, p<0.001
Eating disorders	0.402, p=0.526	0.008, p=0.928	1.770, p=0.183
Personality disorders	7.352, p=0.007	0.144, p=0.705	1.504, p=0.220
Insomnia and parasomnia	42.874, p<0.001	34.589, p<0.001	46.933, p<0.001
Suicidal ideation	2.492, p=0.114	3.588, p=0.058	0.464, p=0.496
Substance use disorders	28.264, p<0.001	20.145, p<0.001	58.537, p<0.001
Self-harm	0.020, p=0.887	1.860, p=0.173	Not reported by TriNetX

Abbreviations: χ^2 values represent the TriNetX proportionality test statistics for the corresponding Cox models. All tests had 1 degree of freedom. A p value < 0.05 indicates evidence against the proportional hazards assumption.

Supplementary Table S2. Baseline characteristics before and after propensity score matching in sensitivity and sex-specific analyses.

Panel A. 30-day post-index lag analysis

Characteristic	HS before PSM (n = 41,272)	Controls before PSM (n = 1,366,092)	SMD	HS after PSM (n = 40,531)	Controls after PSM (n = 40,531)	SMD
Age at index, mean (SD)	34.5 (14.9)	47.5 (22.7)	0.677	34.5 (14.9)	35.1 (15.5)	0.035
Female sex, n (%)	29,926 (73.8)	703,687 (52.6)	0.450	29,901 (73.8)	30,006 (74.0)	0.006

Characteristic	HS before PSM (n = 41,272)	Controls before PSM (n = 1,366,092)	SMD	HS after PSM (n = 40,531)	Controls after PSM (n = 40,531)	SMD
Male sex, n (%)	10,630 (26.2)	633,694 (47.4)	0.450	10,630 (26.2)	10,525 (26.0)	0.006
White race, n (%)	16,008 (39.5)	849,565 (63.5)	0.496	16,008 (39.5)	15,973 (39.4)	0.002
Black or African American race, n (%)	13,523 (33.3)	148,206 (11.1)	0.556	13,499 (33.3)	13,562 (33.5)	0.003
Other race, n (%)	2,700 (6.7)	75,774 (5.7)	0.041	2,700 (6.7)	2,710 (6.7)	0.001
Not Hispanic or Latino ethnicity, n (%)	27,467 (67.7)	902,972 (67.5)	0.004	27,443 (67.7)	27,416 (67.6)	0.001
Unknown ethnicity, n (%)	8,649 (21.3)	353,594 (26.4)	0.120	8,648 (21.3)	8,723 (21.5)	0.005
Tobacco use (Z72.0), n (%)	439 (1.1)	7,065 (0.5)	0.062	438 (1.1)	489 (1.2)	0.012
Personal history of nicotine dependence (Z87.891), n (%)	1,226 (3.0)	80,840 (6.0)	0.146	1,226 (3.0)	1,120 (2.8)	0.016
Crohn disease (K50), n (%)	661 (1.6)	5,762 (0.4)	0.119	636 (1.6)	662 (1.6)	0.005
Ulcerative colitis (K51), n (%)	363 (0.9)	8,188 (0.6)	0.033	355 (0.9)	372 (0.9)	0.004
Overweight and obesity (E66), n (%)	8,941 (22.0)	231,964 (17.3)	0.118	8,933 (22.0)	8,818 (21.8)	0.007
Diabetes mellitus (E08–E13), n (%)	3,719 (9.2)	137,056 (10.2)	0.036	3,717 (9.2)	3,764 (9.3)	0.004
Pain, not elsewhere classified (G89), n (%)	2,617 (6.5)	131,933 (9.9)	0.125	2,616 (6.5)	2,640 (6.5)	0.002
Essential hypertension (I10), n (%)	5,773 (14.2)	414,261 (31.0)	0.409	5,773 (14.2)	5,589 (13.8)	0.013
Chronic kidney disease (N18), n (%)	859 (2.1)	57,547 (4.3)	0.124	857 (2.1)	790 (1.9)	0.012

Panel B. Most recent 5-year analysis

Characteristic	HS before PSM (n = 47,080)	Controls before PSM (n = 1,381,359)	SMD	HS after PSM (n = 46,313)	Controls after PSM (n = 46,313)	SMD
Age at index, mean (SD)	34.6 (14.9)	47.7 (22.6)	0.681	34.6 (14.9)	35.0 (15.3)	0.026
Female sex, n (%)	34,351 (74.1)	712,349 (52.7)	0.456	34,300 (74.1)	34,320 (74.1)	0.001
Male sex, n (%)	12,013 (25.9)	640,299 (47.3)	0.456	12,013 (25.9)	11,993 (25.9)	0.001
White race, n (%)	17,763 (38.3)	858,944 (63.5)	0.521	17,763 (38.4)	17,710 (38.2)	0.002
Black or African American race, n (%)	16,691 (36.0)	152,679 (11.3)	0.608	16,640 (35.9)	16,745 (36.2)	0.005
Other race, n (%)	2,700 (5.8)	75,774 (5.6)	0.010	2,700 (5.8)	2,746 (5.9)	0.004
Not Hispanic or Latino ethnicity, n (%)	32,620 (70.4)	917,311 (67.8)	0.055	32,573 (70.3)	32,564 (70.3)	<0.001

Characteristic	HS before PSM (n = 47,080)	Controls before PSM (n = 1,381,359)	SMD	HS after PSM (n = 46,313)	Controls after PSM (n = 46,313)	SMD
Unknown ethnicity, n (%)	8,963 (19.3)	354,291 (26.2)	0.164	8,961 (19.3)	9,026 (19.5)	0.004
Tobacco use (Z72.0), n (%)	409 (0.9)	6,443 (0.5)	0.049	409 (0.9)	407 (0.9)	<0.001
Personal history of nicotine dependence (Z87.891), n (%)	1,349 (2.9)	76,755 (5.7)	0.137	1,349 (2.9)	1,228 (2.7)	0.016
Crohn disease (K50), n (%)	735 (1.6)	5,216 (0.4)	0.122	684 (1.5)	725 (1.6)	0.007
Ulcerative colitis (K51), n (%)	381 (0.8)	7,599 (0.6)	0.031	367 (0.8)	365 (0.8)	<0.001
Overweight and obesity (E66), n (%)	9,157 (19.8)	223,483 (16.5)	0.084	9,145 (19.7)	9,141 (19.7)	<0.001
Diabetes mellitus (E08–E13), n (%)	4,084 (8.8)	133,935 (9.9)	0.038	4,082 (8.8)	4,065 (8.8)	0.001
Pain, not elsewhere classified (G89), n (%)	2,680 (5.8)	123,712 (9.1)	0.128	2,675 (5.8)	2,677 (5.8)	<0.001
Essential hypertension (I10), n (%)	6,200 (13.4)	410,317 (30.3)	0.419	6,200 (13.4)	6,030 (13.0)	0.011
Chronic kidney disease (N18), n (%)	990 (2.1)	56,998 (4.2)	0.119	989 (2.1)	914 (2.0)	0.011

Panel C. Sex-specific HS analysis

Characteristic	Female HS before PSM (n = 29,059)	Male HS before PSM (n = 10,346)	SMD	Female HS after PSM (n = 10,119)	Male HS after PSM (n = 10,119)	SMD
Age at index, mean (SD)	33.3 (14.3)	37.5 (15.9)	0.274	37.3 (15.7)	37.4 (15.8)	0.002
Female sex, n (%)	28,544 (100.0)	0 (0.0)	—	10,119 (100.0)	0 (0.0)	—
Male sex, n (%)	0 (0.0)	10,166 (100.0)	—	0 (0.0)	10,119 (100.0)	—
White race, n (%)	10,984 (38.5)	3,972 (39.1)	0.012	3,942 (39.0)	3,959 (39.1)	0.003
Black or African American race, n (%)	10,324 (36.2)	2,612 (25.7)	0.228	2,596 (25.7)	2,610 (25.8)	0.003
Other race, n (%)	1,901 (6.7)	688 (6.8)	0.004	688 (6.8)	686 (6.8)	0.001
Not Hispanic or Latino ethnicity, n (%)	19,449 (68.1)	6,425 (63.2)	0.104	6,414 (63.4)	6,398 (63.2)	0.003
Unknown ethnicity, n (%)	5,775 (20.2)	2,742 (27.0)	0.159	2,729 (27.0)	2,724 (26.9)	0.001
Tobacco use (Z72.0), n (%)	252 (0.9)	166 (1.6)	0.067	140 (1.4)	155 (1.5)	0.012

Characteristic	Female HS before PSM (n = 29,059)	Male HS before PSM (n = 10,346)	SMD	Female HS after PSM (n = 10,119)	Male HS after PSM (n = 10,119)	SMD
Personal history of nicotine dependence (Z87.891), n (%)	759 (2.7)	387 (3.8)	0.065	340 (3.4)	372 (3.7)	0.017
Crohn disease (K50), n (%)	432 (1.5)	192 (1.9)	0.029	143 (1.4)	192 (1.9)	0.038
Ulcerative colitis (K51), n (%)	250 (0.9)	87 (0.9)	0.002	77 (0.8)	87 (0.9)	0.011
Overweight and obesity (E66), n (%)	6,741 (23.6)	1,698 (16.7)	0.173	1,561 (15.4)	1,693 (16.7)	0.036
Diabetes mellitus (E08–E13), n (%)	2,413 (8.5)	1,126 (11.1)	0.088	963 (9.5)	1,101 (10.9)	0.045
Pain, not elsewhere classified (G89), n (%)	1,889 (6.6)	557 (5.5)	0.048	521 (5.1)	555 (5.5)	0.015
Essential hypertension (I10), n (%)	3,640 (12.8)	1,762 (17.3)	0.128	1,617 (16.0)	1,720 (17.0)	0.027
Chronic kidney disease (N18), n (%)	448 (1.6)	359 (3.5)	0.125	291 (2.9)	332 (3.3)	0.023

Abbreviations: HS, hidradenitis suppurativa; PSM, propensity score matching; SD, standard deviation; SMD, standardized mean difference. S2 denotes the 30-day post-index lag analysis; S3 denotes restriction to patients indexed within the most recent 5-year period. In the female vs male analysis, sex defined the exposure groups and was not treated as a covariate for balance assessment.

Supplementary Table S3. Event counts and absolute risks for incident psychiatric outcomes in the primary and sensitivity analyses.

Outcome	Analysis	HS events / patients (%)	Control events / patients (%)
Any psychiatric disorder	S1	4,331 / 35,116 (12.33%)	2,030 / 35,116 (5.78%)
Any psychiatric disorder	S2	4,928 / 40,381 (12.20%)	2,367 / 40,498 (5.84%)
Any psychiatric disorder	S3	5,664 / 46,313 (12.23%)	2,696 / 46,313 (5.82%)
Severe psychiatric illness	S1	202 / 35,116 (0.58%)	40 / 35,116 (0.11%)
Severe psychiatric illness	S2	225 / 40,525 (0.56%)	59 / 40,531 (0.15%)
Severe psychiatric illness	S3	254 / 46,313 (0.55%)	59 / 46,313 (0.13%)
Depression	S1	1,626 / 37,964 (4.28%)	662 / 37,964 (1.74%)
Depression	S2	1,797 / 40,493 (4.44%)	755 / 40,527 (1.86%)
Depression	S3	2,034 / 46,313 (4.39%)	822 / 46,313 (1.77%)
Anxiety disorders	S1	2,549 / 37,964 (6.71%)	1,194 / 37,964 (3.15%)

Outcome	Analysis	HS events / patients (%)	Control events / patients (%)
Anxiety disorders	S2	2,789 / 40,470 (6.89%)	1,359 / 40,515 (3.35%)
Anxiety disorders	S3	3,151 / 46,313 (6.80%)	1,518 / 46,313 (3.28%)
Bipolar disorder and manic episodes	S1	147 / 37,964 (0.39%)	24 / 37,964 (0.06%)
Bipolar disorder and manic episodes	S2	155 / 40,526 (0.38%)	29 / 40,531 (0.07%)
Bipolar disorder and manic episodes	S3	179 / 46,313 (0.39%)	35 / 46,313 (0.08%)
Psychotic disorders	S1	88 / 37,964 (0.23%)	26 / 37,964 (0.07%)
Psychotic disorders	S2	94 / 40,530 (0.23%)	36 / 40,531 (0.09%)
Psychotic disorders	S3	100 / 46,313 (0.22%)	32 / 46,313 (0.07%)
Eating disorders	S1	98 / 37,964 (0.26%)	41 / 37,964 (0.11%)
Eating disorders	S2	106 / 40,530 (0.26%)	42 / 40,529 (0.10%)
Eating disorders	S3	111 / 46,313 (0.24%)	46 / 46,313 (0.10%)
Personality disorders	S1	75 / 37,964 (0.20%)	17 / 37,964 (0.04%)
Personality disorders	S2	85 / 40,531 (0.21%)	22 / 40,531 (0.05%)
Personality disorders	S3	89 / 46,313 (0.19%)	17 / 46,313 (0.04%)
Insomnia and parasomnia	S1	2,360 / 35,311 (6.68%)	2,282 / 35,404 (6.45%)
Insomnia and parasomnia	S2	2,521 / 37,552 (6.71%)	2,370 / 37,650 (6.29%)
Insomnia and parasomnia	S3	2,991 / 43,076 (6.94%)	2,808 / 43,039 (6.52%)
Suicidal ideation	S1	118 / 37,938 (0.31%)	44 / 37,950 (0.12%)
Suicidal ideation	S2	129 / 40,504 (0.32%)	54 / 40,520 (0.13%)
Suicidal ideation	S3	148 / 46,283 (0.32%)	49 / 46,304 (0.11%)
Substance use disorders	S1	1,568 / 37,964 (4.13%)	431 / 37,964 (1.14%)
Substance use disorders	S2	1,705 / 40,477 (4.21%)	453 / 40,522 (1.12%)
Substance use disorders	S3	2,008 / 46,313 (4.34%)	558 / 46,313 (1.20%)
Self-harm	S1	19 / 35,110 (0.05%)	13 / 35,114 (0.04%)
Self-harm	S2	20 / 40,524 (0.05%)	11 / 40,527 (0.03%)
Self-harm	S3	Not reported	Not reported

Abbreviations: HS, hidradenitis suppurativa; S1, primary analysis; S2, 30-day post-index lag analysis; S3, most recent 5-year analysis.

Note: Percentages were calculated as events divided by the number of patients included in the corresponding outcome-specific analysis after exclusion of patients with that outcome before the analysis time window. Self-harm in S3 was not reported because TriNetX did not generate a reliable estimate for this outcome.
